# Early Polylysine Release from Dental Composites and Its Effects on Planktonic *Streptococcus mutans* Growth

**DOI:** 10.3390/jfb11030053

**Published:** 2020-07-27

**Authors:** Nikos N. Lygidakis, Elaine Allan, Wendy Xia, Paul F. Ashley, Anne M. Young

**Affiliations:** 1 Unit of Paediatric Dentistry, Department of Craniofacial Growth and Development, UCL Eastman Dental Institute, London WC1X 8LD, UK; nikos.lygidakis@gmail.com (N.N.L.); p.ashley@ucl.ac.uk (P.F.A.); 2 Division of Microbial Diseases, UCL Eastman Dental Institute, London WC1X 8LD, UK; e.allan@ucl.ac.uk; 3 Department of Biomaterials and Tissue Engineering, UCL Eastman Dental Institute, London WC1X 8LD, UK; wendy.xia2011@yahoo.co.uk

**Keywords:** polylysine, antibacterial, dental composites

## Abstract

The study aim was to assess the effect of incorporating polylysine (PLS) filler at different mass fractions (0.5, 1 and 2 wt%) on PLS release and *Streptococcus mutans* planktonic growth. Composite containing PLS mass and volume change and PLS release upon water immersion were assessed gravimetrically and via high-performance liquid chromatography (HPLC), respectively. Disc effects on bacterial counts in broth initially containing 8 × 10^5^ versus 8 × 10^6^ CFU/mL *Streptococcus mutans* UA159 were determined after 24 h. Survival of sedimented bacteria after 72 h was determined following LIVE/DEAD staining of composite surfaces using confocal microscopy. Water sorption-induced mass change at two months increased from 0.7 to 1.7% with increasing PLS concentration. Average volume increases were 2.3% at two months whilst polylysine release levelled at 4% at 3 weeks irrespective of composite PLS level. Early percentage PLS release, however, was faster with higher composite content. With 0.5, 1 and 2% polylysine initially in the composite filler phase, 24-h PLS release into 1 mL of water yielded 8, 25 and 93 ppm respectively. With initial bacterial counts of 8 × 10^5^ CFU/mL, this PLS release reduced 24-h bacterial counts from 10^9^ down to 10^8^, 10^7^ and 10^2^ CFU/mL respectively. With a high initial inoculum, 24-h bacterial counts were 10^9^ with 0, 0.5 or 1% PLS and 10^7^ with 2% PLS. As the PLS composite content was raised, the ratio of dead to live sedimented bacteria increased. The antibacterial action of the experimental composites could reduce residual bacteria remaining following minimally invasive tooth restorations.

## 1. Introduction

Dental caries is one of the most common diseases affecting humans. Traditionally, treatment involved the removal of all the affected tooth structure followed by replacement with amalgam. Silver mercury amalgam is a highly effective restorative material due to both its good mechanical and antibacterial properties. Following the Minamata agreement in 2013 on global mercury reduction signed by 140 counties, amalgam is being phased out. In Europe, amalgam use in primary teeth was banned in 2018 [1].

Modern dentistry advocates for the removal of highly infected surface dentine but not underlying demineralized and less infected dentine that may be close to the pulp [2]. Effective cavity sealing with Glass Ionomer Cement (GIC) restorations can reduce any underlying residual bacterial contamination. It can also encourage remineralization but have insufficient strength for larger cavities.

Conversely, dental composites have greater strength, but their placement is complex and their technique sensitive. Multiple adhesion-promoting steps are required, in addition to material placement and light curing in several increments to reduce polymerization shrinkage consequences [3]. Composite failure is usually due to secondary caries at the restoration margins. This is attributed to a combination of residual bacteria, shrinkage and enzyme-activated degradation of demineralized dentine, which may compromise the bonding interface, enabling further bacterial penetration and growth [4]. A composite material that could provide surface release of antibacterial and remineralizing agents upon damage of the tooth/restoration interface might therefore be beneficial.

Antibacterial components such as chlorhexidine, fluoride, quaternary ammonium methacrylate, silver and triclosan have previously been added to dental composites [5,6,7,8,9]. Polylysine (PLS) is an alternative antibacterial agent that has been included in composites more recently. Polylysine-greater eukaryotic cell-compatibility may address the limited biocompatibility of some other antibacterial agents [10,11]. Studies suggest electrostatic adsorption of polylysine on bacterial surfaces, and its abnormal distribution within their cytoplasm, leads to their cell damage [12,13]. Polylysine, however, is generally recognized as safe (GRAS). It is a natural polypeptide that is biodegradable, water-soluble, nontoxic and edible. Additionally, polylysine is extensively used in eukaryotic cell culture to promote cell attachment and as a food preservative [12,14]. In the human body, polylysine degrades harmlessly to give the essential amino acid, lysine [12,14]. Furthermore, polylysine addition into calcium phosphate-containing composites can enhance apatite precipitation on their surfaces from simulated body fluid. Polylysine may therefore also enhance composite remineralizing potential [10].

Whilst many properties of polylysine-containing dental composites have now been investigated [10,11], publications on their actions against bacteria are limited. In this study, therefore, the ability of composites with increasing levels of polylysine to affect bacterial growth was investigated. Effects on planktonic bacteria are assessed to model the feasibility of surface polylysine release reducing bacterial microleakage in gaps at the tooth restoration interface. Polylysine release kinetics are provided to help explain the varying levels of antibacterial action. Potential polylysine addition and release effects on set material stability are monitored through mass and volume changes in water.

## 2. Materials and Methods

### 2.1. Components

The resin matrix was prepared using Urethane Dimethacrylate (UDMA) (DMG, Hamburg, Germany) as a base monomer and PolyPropylene Glycol Dimethacrylate (PPGDMA) (Polysciences, Inc., Warrington, FL, USA) as a diluent monomer. 4-methacryloxyethyl trimellitate anhydride (4-META) (Polysciences, Inc., Warrington, FL, USA) and Camphorquinone (CQ) (DMG, Hamburg, Germany) were added at low level. The filler consisted of two different sizes of aluminosilicate glass of 7 μm and 0.7 μm (DMG, Hamburg, Germany), fumed silica (Aerosil OX 50, Evonik, Essen, Germany), monocalcium phosphate monohydrate (MCPM) (Himed, Old Bethpage, NY, USA) and polylysine (PLS) (Handary, Brussels, Belgium).

UDMA is a common base monomer, that has been used as an alternative to bisphenol A-glycidyl methacrylate (Bis-GMA) [15]. In previous studies, PPGDMA, a diluent monomer that can be used instead of triethylene glycol dimethacrylate (TEGDMA), enhanced paste stability, increased light activated polymerization and reduced associated shrinkage [11]. 4-META is an adhesion-promoting monomer [16]. Monocalcium phosphate monohydrate (MCPM) particles added to composites can promote water sorption and react to produce brushite crystals of greater volume thereby giving expansion to compensate polymerisation shrinkage. Furthermore, release of its phosphate ions can promote apatite precipitation from simulated dentinal fluid to remineralize dentine [17].

### 2.2. Paste Preparation

Four formulations with 1 variable (PLS) were prepared. The resin (liquid) phase consisted of UDMA (72 wt%), PPGDMA (24 wt%), 4-META (3 wt%), and CQ (1 wt%) [11]. This was prepared by mixing the components and stirring for 24 h at room temperature on a magnetic stirrer hot plate (Jeo Tech) until a clear liquid was achieved. The filler phase contained glass of 7 μm, 0.7 μm and fumed silica in the ratio 6:3:1. 10 wt% MCPM was added to the filler as in F2 in previous work [11] but the tricalcium phosphate was removed. Additionally, the filler PLS level was varied (0, 0.5, 1 or 2 wt%) instead of being fixed at 2 wt%. Filler: resin were mixed in the weight ratio 5:1 for 45 s at 3500 rpm using a centrifugal mixer (Speedmixer, Hauschild Engineering, Hamm, Germany). This high ratio produced pastes with a consistency comparable with the commercial packable composite Filtek Z250 (3M, Bracknell, UK) used as an additional control in antibacterial studies.

### 2.3. Composite Discs Preparation

**Figure 1:**
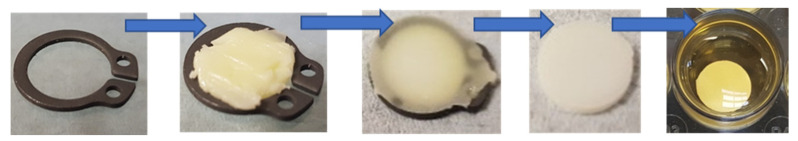
Preparation of composite disc and disc placed in well.

Disc-shaped specimens were formed by applying the composite pastes within metal circlips with internal diameter 10 mm and thickness of 1 mm and pressing them between two sheets of acetate (Figure 1). The specimens were then photopolymerized using a blue light-emitting diode curing unit with a wavelength of 450–470 nm and power of 1100–1300 mW/cm^2^ (Demi Plus, Kerr Dental, Bioggio, Switzerland) with the tip in contact with the acetate sheet. The curing duration was 40 s on each side of each disc. This method gives 72% monomer conversion for the formulations [11]. The discs were then removed from the circlip, any excesses were trimmed with a no.11 blade, and stored at room temperature in the dark until required.

### 2.4. Mass and Volume Change

The mass and volume change of set discs versus time in deionised water (DW) were determined using a density kit and four-figure digital balance (AG204, Mettler, Toledo, OH, USA) according to the ISO 17304:2013. 1% sodium dodecyl sulphate (Sigma-Aldrich, Gillingham, UK) in DW was used as the buoyancy medium.

Discs (*n* = 3), prepared as above, were immersed in 1 mL of DW in individual sterilin tubes at 23 °C. At 1, 3, 6 h, 1, 2, 5, 7 days, 2, 4 and 8 weeks, the discs were removed from the solution, their surfaces blot dried using absorbent paper, weighed in air and in the buoyancy medium and then placed into new tubes with fresh 1 mL of DW. Initial mass and volume were calculated by extrapolation of early data versus square root of time to zero. Mass and volume change were then calculated as percentages of original mass as shown in detail in previous publications [10,11].

### 2.5. Polylysine Release

High-performance liquid chromatography (HPLC) (Shimadzu corporation, Kyoto, Japan) was used to measure polylysine release from composite discs. The composite discs (*n* = 3) were prepared as above, weighed and then immersed in 1 mL deionized water (DW) in individual sterilin tubes. The discs were removed from the DW and placed in a new tube with 1 mL of fresh DW at 1, 3, 6 h, 1, 2, 5, 7 days and 3 weeks. The storage solutions were stored at 23 °C prior to analysis.

A normal phase column in hydrophilic interaction liquid chromatography (HILIC) mode was used. The mobile phase was 50 vol% acetonitrile in DW with 0.1 vol% of phosphoric acid (97%) added. Flow rate, run time, temperature, and UV detection wavelength were 1.0 mL/min, 43 min, 30 °C and 210 nm respectively. PLS solutions of 10 to 100 ppm were used to generate a calibration curve which was then employed to determine PLS concentrations in each storage solution. Cumulative PLS versus time, as a percentage of that calculated to be in the original specimen, was then determined [10].

### 2.6. Bacterial Growth Inhibition

A single colony of *S. mutans* UA159 was inoculated into brain heart infusion broth (BHI, Oxoid, Basingstoke, UK) and incubated statically at 37 °C for 16 h in air enriched with 5% CO_2_. The culture was diluted in BHI broth to generate an inoculum of 8 × 10^5^ and 8 × 10^6^ CFU/mL which was confirmed by viable counting.

On the day of each experiment the discs (*n* = 3) were placed in a custom made ultraviolet light box and irradiated for 30 min on each side to ensure sterility. Decontamination was verified by plating on agar. The discs were then placed into a 24 well plate as seen in Figure 1. 1 mL of the *S. mutans* inoculum was added to each well and the plates placed in the incubator on a shaking tray at 200 rpm at 37 °C in air. At 24 h, the cultures were 10-fold serially diluted and plated out on BHI agar (Oxoid, Basingstoke, UK). Bacterial colonies were counted after 3 days of incubation at 37 °C in 5% CO_2_.

### 2.7. Surface Bacteria Observations

To assess viability of attached bacteria on material surfaces, composite discs (*n* = 2) were immersed in an *S. mutans* suspension with concentration of 5 × 10^6^ CFU/mL and incubated statically for 72 h in air at 37 °C. The discs were removed and gently immersed in fresh BHI broth to remove unattached bacteria before placing in a clean well plate for staining using LIVE/DEAD Viability kit (Thermofisher Scientific, Loughborough, UK). Confocal laser scanning microscopy (Radiance 2100, Biorad, Hercules, CA, USA) with an objective lens of 10×–20× magnification was used to visualize bacteria. Images were saved and processed using Lasersharp 2000 (Biorad, Hercules, CA, USA) and ImageJ (ImageJ Developers).

### 2.8. Statistical Analysis

Data analysis was undertaken for volumetric analysis, polylysine release and bacterial viable counts using Analysis of Variance (ANOVA) and SPSS (IBM, Armonk, NY, USA) with *p* < 0.05 considered significant. To perform multiple comparisons of multiple formulations, the Bonferroni adjustment was used.

## 3. Results

### 3.1. Mass and Volume

**Figure 2:**
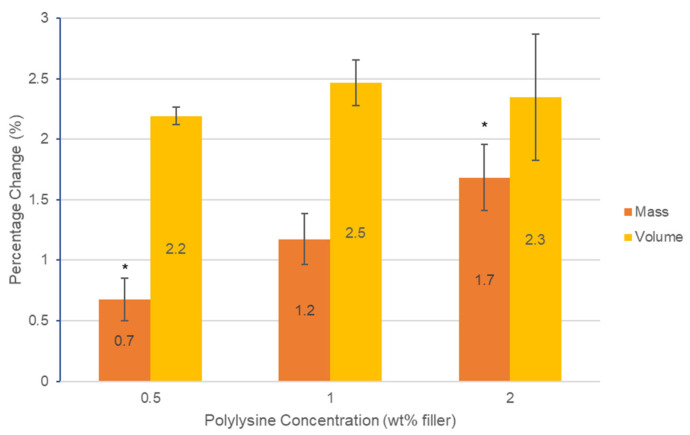
Graph of percentage change in mass and volume of the three formulations containing polylysine after 2 months. Error bars = st. dev. (*n* = 3). * indicates significance between results (*p* < 0.05).

Mass and volume change both increased proportional to the square root of time. Final mass change at 2 months increased linearly with polylysine content from 0.7 to 1.7 wt% (RSQ = 0.96). Final volume change, however, was not significantly affected by PLS level (*p* > 0.05) and was between 2.2 and 2.5 vol% (Figure 2).

### 3.2. Polylysine Release

**Figure 3:**
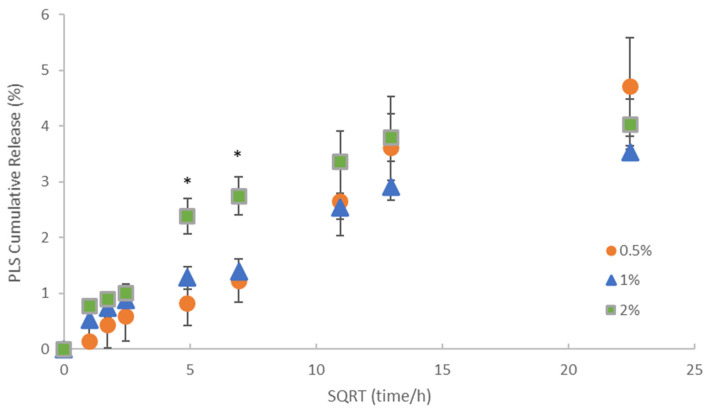
Cumulative percentage release of polylysine (PLS) versus the square root (SQRT) of time. Error bars = st. dev (*n* = 3). * indicates that formulation with 2% PLS has significantly higher percentage release at 24 and 48 h but not at early or later times.

**Figure 4:**
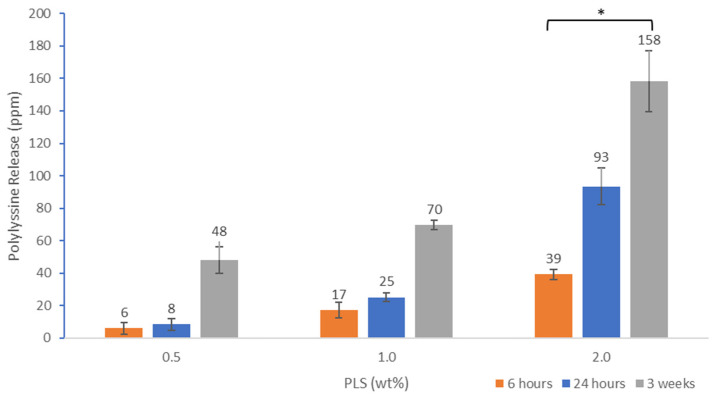
Cumulative polylysine release after 6 h, 24 h and 3 weeks at 23 °C for formulations with 0.5, 1 or 2% PLS. Error bars = st. dev (*n* = 3). * indicates 2% PLS containing formulation has significantly higher release at all time points compared with the other 2 formulations.

Cumulative polylysine release was initially proportional to the square root of time but then levelled between 2 and 3 weeks (Figure 3). Cumulative percentage release was higher with 2% PLS at 1 and 2 days at 2.4 and 2.7% but within experimental error independent of concentration and 4% by 3 weeks. In Figure 4, the percentage release is converted to provide a calculated total concentration per disc that would have been released into 1 mL of storage solution by 6 h, 24 h and three weeks. At 3 weeks, the PLS release in grams was proportional to the level in the filler. At earlier time points, however, doubling the filler PLS content more than doubled the release in grams (see Figure 4).

### 3.3. Determination of Antibacterial Activity

**Figure 5:**
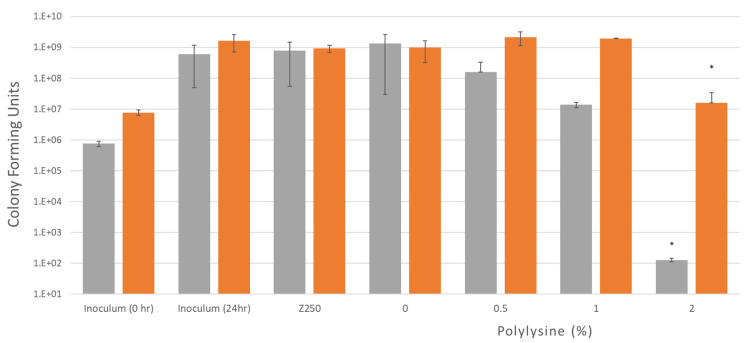
Bacterial growth after 24 h with two different inoculum concentrations of *S. mutans*, 8 × 10^5^ and 8 × 10^6^ CFU/mL. Four different formulations and a commercial composite were tested. Plates with discs and inoculum were incubated in air for 24 h at 37 °C while being shaken. Error bars = st. dev (*n* = 3), * indicates 2% is significantly different when compared to other formulations.

Colony forming units are shown in Figure 5, after 24 h incubation in air with different composite formulations for two different initial inoculum bacterial levels. Irrespective of initial inoculum level, CFU increased to 10^9^ for all controls with no PLS. This was also observed with 0.5% and 1% PLS with the higher initial inoculum. Conversely, 0.5 and 1% PLS in the composite filler caused a 90 and 99% reduction in CFU with the lower initial inoculum concentration. With 2% PLS, the 24 h CFU count was down to 10^7^ and 10^2^ with high versus low initial inoculum levels, respectively. The 2% PLS formulations had statistically significantly less bacteria at 24 h when compared to all other composites at both inoculum concentrations.

### 3.4. Confocal Laser Scanning Microscopy

**Figure 6:**
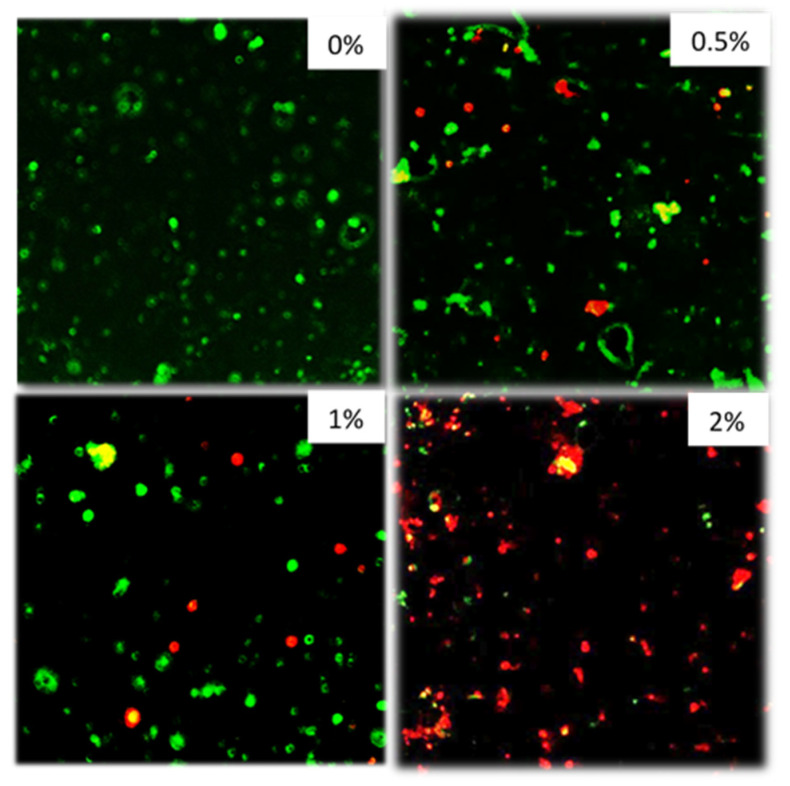
From top left to bottom right 0, 0.5, 1, 2% formulations. Inoculum: *S. mutans* 5 × 10^6^ CFU/mL. Plates with discs and inoculum were incubated for 72 h in air at 37 °C before staining. Stained with Live/Dead staining. (*n* = 2).

Figure 6 shows confocal images of composite surfaces stained with live/dead stain. The bacteria do not form a biofilm due to the lack of sucrose but are seen as individual bacteria on the material surface. As the concentration of polylysine in the composite disc increases, an increase in the proportion of dead bacteria is seen.

## 4. Discussion

### 4.1. Mass and Volume Change

Dental restorations are exposed to fluids from the oral cavity continuously. Any dental composite will have micro voids after the setting reaction has taken place. When immersed in solution (i.e., deionized water), over time, the water will be absorbed by the voids in the composite as well as the composite matrix phase and the mass and volume will increase. Excessive expansion can result in a reduction in mechanical properties [18].

Mass and volume changes were undertaken for 2 months, as previous work has shown they tend to level off after this time [19]. With low PLS, the volume change, approximately double the mass change, suggests water is mostly expanding the matrix phase [19]. This is a consequence of the composite density being double that of water. With higher PLS, the mass increase, more comparable with the volume change, suggests there are more pores being filled by water which would cause mass increase but no expansion. The porosity may be caused by poor wetting and dispersion of the hydrophilic PLS particles by the monomer phase. The lack of change in the volume with increasing PLS level suggests it has minimal effect on the surrounding matrix expansion. The final levels of mass and volume change with 2% PLS (1.7 wt% and 2.3 vol%) are approximately two thirds of those seen with an earlier similar formulation (F2) (2.5 wt% and 3.5 vol%) that had an additional 10 wt% tricalcium phosphate [11]. This suggests the mass and volume changes are related to the total calcium phosphate levels in these composites. A previous study has shown that the commercial composite Z250 shows an increase in mass and volume after 7 weeks of 1.1 and 1.6% respectively [11]. The mass and volume changes of the experimental composites were comparable or above those observed for Z250.

### 4.2. Antibacterial Activity and Polylysine Release

Polylysine has been added to dental composites in previous research [10,11], but reports on the optimum concentration have not been carried out. This study attempts to replicate the conditions at the interface between the composite and the sealed affected dentine in which sucrose will be excluded. In this situation, the formation of biofilm on the discs was not observed. One recent study demonstrated that polylysine had satisfactory antibacterial efficacy against *S. mutans* in a liquid culture medium and as an application on biofilm–dentin surfaces. The study demonstrated that the susceptibility of microorganisms to polylysine was dependent on polylysine concentrations [20]. Additionally, it showed that polylysine can kill and inhibit the growth of the periodontal pathogen *Porphyromonas gingivalis* [20].

Increased polylysine release from the composite can have a negative effect on the mechanical properties of the material [11], so the minimum amount to cause bacterial kill would need to be used. This is a common issue for formulations with a released soluble antibacterial agent [5,6]. Other antibacterial agents, such as quaternary ammonium methacrylate, are non-released and their effect on physical properties is less evident [8,21]. Additionally, their surface antibacterial properties could be more stable over time. Surface antibacterial benefits, however, may be neutralized by a biofilm or adhesive covering. Conversely, the benefit of high early polylysine release is a greater chance of an effective reduction in residual bacteria deep within minimally excavated cavities. The above studies suggest, however, that higher levels of polylysine release may be required for highly infected cavities.

Previous experiments have shown greater early burst release and a higher release over time from a 2.5% PLS formulation with approximately 5.5% after 24 h and 9% at 3 weeks of being observed. As this was not previously affected by monocalcium and tricalcium phosphate addition, the differences may instead be due to the use of the more standard TEGDMA monomer. This can cause lower conversions [10] than seen with PPGDMA [11]. In this new study, the composite formulation with 2% PLS released 2.4% of polylysine in the first 24 h and after 3 weeks this had increased to just 4%. Initial polylysine release, proportional to the square root of time with no early burst release, is more in agreement with a recent report using the same monomer system as this study [22]. The work showed that increasing the polylysine concentration from 4 to 6 and then 8 wt% of the filler caused final release percentages to level at values of 13, 28 and 42% after 1, 2 and 3 months respectively [22]. This was attributed to surface release from layers of 65, 140 and 210 micron thick. The PLS release levelling between 1 and 3 weeks in this study fits with this trend. Given that the sample thickness is 100 microns, a final release of 4% suggests the PLS release may be from surface layers of just 20 microns thick.

Due to the initially higher concentration of PLS, the release in grams is higher as the concentration of polylysine in the formulations increased from 0.5 to 2%. The Minimum Inhibitory Concentration of polylysine on *S. mutans* in air with inoculum density of 5 × 10^5^ CFU/mL is 20 μg/mL [23]. After 24 h, the 0.5%, 1% and 2% formulations had an average (*n* = 3) of 8, 25 and 93 μg/mL of polylysine released. This could explain why the 1% and 2% formulations inhibit growth in air conditions as they release more than 20 μg/mL of polylysine. Conversely, the 0.5% formulations which released less than 20 μg/mL did not inhibit growth. It is therefore expected that higher PLS concentrations will be required to inhibit growth of higher inoculum concentrations. This was also demonstrated in a recent study [20].

The aim of using Confocal was to visualize the live/dead state of bacteria sedimented on the composite disc surfaces. In many other studies, additional sucrose is added to promote biofilm formation [8,9]. The method chosen in this study with no additional sucrose, however, better replicates the environment beneath the restoration after effective sealing. With these conditions, largely only well-spaced individual bacteria were seen on the material surfaces and not biofilms. With the 0.5% and 1% PLS discs, some dead bacteria were present on disc surfaces at 72 h, whereas on the 2% PLS discs, the majority were dead. At the high level of initial inoculum chosen for the confocal study, the 2% PLS disc prevents any change in bacterial numbers in suspension at 24 h indicating bacterial increase is exactly balanced by killing. It is possible, therefore, that dead bacteria on the composite surfaces were killed when they made contact with remaining PLS on the composite surfaces or through more prolonged and increasing contact with PLS in the suspension.

## 5. Conclusions

Increasing polylysine concentration from 0.5% to 2% in composite discs leads to an increase in mass but not volume change over three weeks. Whilst the PLS-release percentage was faster in the first 24 h with 2% in the filler, the final percentage release was not affected by increasing composite PLS level. With 2% PLS, the amount released into 1 mL of broth in 24 h was sufficient to display significantly reduced bacterial levels when the initial inoculum level was at 8 × 10^5^, but their growth in number was only inhibited when the initial bacterial counts were raised 10-fold. With an initial inoculum of 5 × 10^6^ bacteria, those detected on the composite surfaces after 72 h were mostly live with 1% or less PLS in the composite but dead with 2% PLS. Higher PLS may enable the greater killing of residual bacteria when composites are used for minimally invasive tooth restorations. Considering the limitations of this study and taking into account work completed in previous studies [24,25,26], future research should include sucrose in the experiments to allow for biofilm growth on the discs to determine the effectiveness of PLS against biofilms, test multispecies biofilms and assess long term polylysine release and how this may affect the integrity of the restoration.

## 6. Patents

The work is covered by the following licensed patent families: Formulations and composites with reactive fillers (US8252851 B2, EP2066703B1, US20100069469, WO2008037991A1), and Formulations and materials with cationic polymers (PCT/GB2014/052349, WO2015015212 A1, EP3027164A1, US20160184190).
